# The Optimization Strategy of the Existing Urban Green Space Soil Monitoring System in Shanghai, China

**DOI:** 10.3390/ijerph18094820

**Published:** 2021-04-30

**Authors:** Weiwei Zhang, Jigang Han, Abiot Molla, Shudi Zuo, Yin Ren

**Affiliations:** 1 Key Laboratory of National Forestry and Grassland Administration on Ecological Landscaping of Challenging Urban Sites, Shanghai Academy of Landscape Architecture Science and Planning, Shanghai 200232, China; zww@shsyky.com; 2 Shanghai Engineering Research Center of Landscaping on Challenging Urban Sites, Shanghai 200232, China; 3 Key Laboratory of Urban Environment and Health, Institute of Urban Environment, Chinese Academy of Sciences, Xiamen 361021, China; sdzuo@iue.ac.cn (S.Z.); yren@iue.ac.cn (Y.R.); 4 University of Chinese Academy of Sciences, Beijing 100049, China

**Keywords:** background value, concentration, prediction accuracy, soil pollution, optimization

## Abstract

High concentrations of potentially toxic elements (PTE) create global environmental stress due to the crucial threat of their impacts on the environment and human health. Therefore, determining the concentration levels of PTE and improving their prediction accuracy by sampling optimization strategy is necessary for making sustainable environmental decisions. The concentrations of five PTEs (Pb, Cd, Cr, Cu, and Zn) were compared with reference values for Shanghai and China. The prediction of PTE in soil was undertaken using a geostatistical and spatial simulated annealing algorithm. Compared to Shanghai’s background values, the five PTE mean concentrations are much higher, except for Cd and Cr. However, all measured values exceeded the reference values for China. Pb, Cu, and Zn levels were 1.45, 1.20, and 1.56 times the background value of Shanghai, respectively, and 1.57, 1.66, 1.91 times the background values in China, respectively. The optimization approach resulted in an increased prediction accuracy (22.4% higher) for non-sampled locations compared to the initial sampling design. The higher concentration of PTE compared to background values indicates a soil pollution issue in the study area. The optimization approach allows a soil pollution map to be generated without deleting or adding additional monitoring points. This approach is also crucial for filling the sampling strategy gap.

## 1. Introduction

The quality of the urban ecosystem depends on the green space soil quality. Soil quality refers to the soil’s ability to ensure biological productivity, maintain environmental quality, and promote organism health functions within the limit of ecosystems [1]. Rapid urbanization, industrialization [2] and greenery development will affect the soil quality in urban areas [3]. Therefore, urban areas become the sources of various pollutant elements that can be accumulated for an extended period of time in the soil [4,5,6]. Studies on the concentrations of potentially toxic elements (PTE) in urban soils, previously known as heavy metals, started in the 1960s and identified massive heavy metals sources of urban soil pollution [4,5]. The origins of PTE in urban soils are natural and anthropogenic. The pedogenesis processes are considered the natural source of PTE in the soil [7]. The anthropogenic factors are the crucial sources of PTE in soils and predominantly result from urban development and urbanization [8], the distribution of vehicles and the types of fuels [9], emission from industries and transportation [10], smelting, manufacturing, mining, and coal-burning [11]. Based on these factors, urban soils are enriched with a high level of PTE compared to threshold values [12,13,14].

Numerous studies about PTE in urban soils have been conducted in many cities around the world, including Glasgow [15], London [16], Hong Kong [17], New Orleans [18], and Oslo [19]. To date, studies on urban areas in North Pakistan [2], Brazilian Amazon [20], South-central Poland [21], Southwest Iran [22], Eastern China [23], Istanbul Turkey [24], Xiangtan Central China [25], and Krakow Poland [26]. These studies showed increased concentrations of PTE in urban soils. High concentrations of PTE, especially in green space soils, create global environmental stress due to the crucial threat to the environment and human health [27]. High concentrations of PTE in the environment are a concern due to their toxicity, persistence, and bioaccumulation, which threaten the health of all living systems [28]. For example, PTE in soil affects the key microbial processes, decreases soil microorganism diversity and activity [29], and impacts the food chain systems in the environment [30]. Health issues associated with PTE are associated with respiration, hand-mouth ingestion, and direct skin contact [31]. Excessive intake can lead to digestive disorders, respiratory diseases, abdominal pain, vomiting, anorexia, burnout, hemolysis, liver, and gallbladder damage [30]. Since urban green spaces are places where local populations can rest, play, and socialize, they often serve as family gathering sites after work [32]. These may favor contact with soil contaminants. Many previous studies focused on industrial areas and zones. Recently, the evaluation of PTE has become a high priority in urban greening space soils [33]. Furthermore, many authors are concerned about analyzing the total contents of PTE compared with background values to evaluate the environmental quality [31,34]. Nevertheless, the analysis of the total concentrations of PTE, compared with threshold values in the soil, may not always be a sufficient strategy for assessment [35,36,37], and in identifying appropriate remediation strategies [38].

Monitoring PTE concentrations with improved prediction accuracy through an optimization strategy is necessary for obtaining reliable results in surveying soil pollution and making sustainable environmental decisions on urban green spaces. Optimization is the process of selecting the optimal sample points and layouts based on the distance between the observation points and the interpolation grid [39]. One useful method using geostatistical tools for sampling design and predicting concentrations of soil pollution is kriging. Kriging not only provides an interpolated concentration map, but it is also useful in linking the prediction variance [40]. Minimizing the mean kriging variance as the objective function is used to optimize the sampling scheme and produce a space-filling distribution over the area of interest [41,42]. This optimization ensures the prediction of soil pollution and fills the sampling strategy gap by perpetuating limited sampling points, without deleting and adding monitoring points using spatial simulated annealing (SSA) algorithm. Such optimization and improved sampling design [39,41,43] are very useful, since no standardized approaches exist for choosing sample size and locations for soil and air pollution [44].

The main objectives of this work were as follows: (i) Evaluate the concentration levels of five PTE (copper (Cu), zinc (Zn), cadmium (Cd), chromium (Cr), and lead (Pb)) in green space soil; (ii) improve the prediction accuracy of the initial sampling design using an optimization strategy, and showing methodological approaches how to generate of soil pollution map without the extra expense. Since the pre-survey launched before found that the urban green soils were mainly dominated by these five elements. The Shanghai municipality started to focused on: (1) Investigating the contamination situation and sources differentiation of the five PTE on greens spaces soils, (2) managing and reducing soil pollution risks on urban parks [45]. 

## 2. Materials and Methods

### 2.1. Study Area

**Figure 1:**
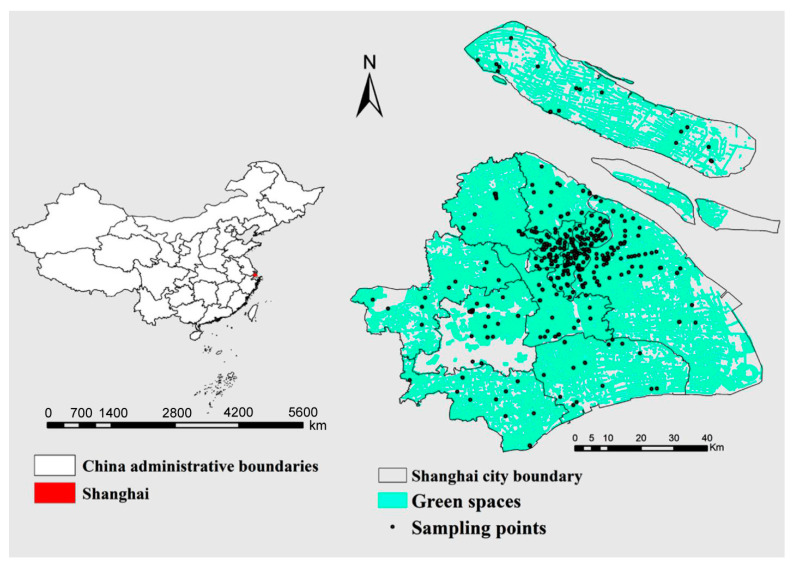
Location of study areas and sampling point’s distribution on urban green spaces.

The study is conducted in Shanghai, one of China’s most highly developed and densely populated cities. It is located at 31.14° N and 121.29° E (Figure 1). Shanghai is one of the most extensive coastal cities in eastern China, which plays a crucial role in its main economic, financial, trade, and shipping center, with the most important industrial centers in China. The town covers about 6340.5 km^2^, of which 6218.65 km^2^ is the land, and the rest is water, and it covers 0.06% of China’s total territory [46]. The soil types mainly include paddy soil, fluvial-aquic soil, and coastal saline soil [46]. The entire green spaces in 2015 were about 3593.5 km^2^ [47]. The city has characterized the subtropical monsoon climate, with an annual mean temperature of 16 °C and yearly average precipitation is approximately 1200 mm. 

### 2.2. Soil Sampling and Chemical Analysis 

A total of 460 surface soil (0–20 cm) samples were collected from different green spaces in 2018. The locations were recorded using a global positioning system (GPS) and displayed in Figure 1. Five random soil samples were collected using a soil corer (2.5 cm diameter) and then pooled into one composite sample. The composite samples were air-dried, cleared of visible plant roots and residues. In order to ensure the complete digestion of soil samples, the air-dried soils were ground and passed through a 0.15 mm nylon mesh sieve. For each sample, 0.5 g of soil was digested with a concentrated mixture of HNO_3_, HF, and HClO_4_ as stated in the EPA 3052 method [48]. Mixed acid digestion makes the soil digestion more complete. Therefore, compared with the aqua regia digestion, the result of mixed acid digestion becomes higher, which is closer to the actual concentrations of PTE in the soil. The five PTE, including Cu, Zn, Cd, Cr, and Pb contents, were measured using Inductively Coupled Plasma Mass Spectrometry (ICP-MS, NexION 300X, Spectralab Scientific Inc. Markham, ON L3R 3V6, Canada). The limit of detection (LOD) and limit of quantification (LOQ) for the different metals were determined. The LOD for analysis of Cr, Cu, Zn, Cd, and Pb were 0.47 mg kg^−1^, 0.25 mg kg^−1^, 0.70 mg kg^−1^, 0.01 mg kg^−1^, and 0.30 mg kg^−1^, respectively. The LOQ of the above five PTE was four times their respective LOD. Certified soils (GSS series, China) were used as standard reference materials to verify the accuracy of the method, and the recovery rate of all measured PTE was 95–105%. All tested glass and blanks were soaked in HNO_3_, rinsed, and Milli-Q water to prevent contamination of the testing instrument.

### 2.3. Geostatistical Methods

Geostatistics is an extension tool in GIS that describes the spatial variation and carries out spatial interpolations [49]. The semivariance function and the kriging interpolations were used to produce the initial interpolation map on green spaces soil [50]. 

Semivariogram is equal to one-half of the expected value of the squared differences between values of X at locations (i) and (i + h) [51],

(1) $$\gamma \left(h\right)=\frac{1}{2m\left(h\right)}\sum_{i=1}^{m\left(h\right)}{\left[Z\left(x_{i}\right)-Z\left(x_{i}+h\right)\right]}^{2}$$

where m(h) is the number of pairs of observations separated by distance h, Z(x_i_) is the sample value of the variable Z at location x_i_, and Z(x_i_ + h) is the sample value of the variable Z at location x_i_ + h.

The ordinary Kriging interpolation is one of the most frequently used geostatistics tools to estimate unknown values using the sample data [52],

(2) $$\hat{z}\left(x_{0}\right)=\overset{n}{\underset{i=0}{\sum }}y_{i}z\left(x_{i}\right)$$

where $\hat{z}$(x_0_) is the value to be estimated at the location of x_0_; and z(x_i_) is the known value at the sampling site x_i_; y_i_ represents constant values of each local neighborhood. While, n represents the number of sites or sampling points within the search neighborhoods used for the estimation.

The existing monitoring points were visualized and analyzed using exploratory spatial data analysis (ESDA) tools. ESDA was used to assess the degree of spatial association and examine how the data are normally distributed [53,54]. The spatial clusters and outliers of existing data sets were identified using Local Moran’s I [55] and Global Moran’s I statistic [56]. 

### 2.4. Prediction Accuracy Improvement Procedures

The prediction accuracy improvements can normally be achieved by optimizing sample locations over the geographical areas [57]. Optimization usually consists of adding, removing, and moving stations or sampling points [58]. One of the optimization algorithms used to add, remove, and transfer stations to generate optimized sampling sizes and designs is called SSA [42]. The SSA algorithm uses the mean kriging variance (MKV) as the objective function to obtain an optimal sample layout. In this case, the initial design was optimized by moving existing spatial points to the given study surface areas using soil Pb data as an example. Sample optimization by SSA also considers the kriging prediction and fitting variogram models [59]. Then, data were log-transformed before spatial optimization analysis was undertaken. The detailed optimization and evaluation techniques were explained as follows.

#### Perturb Initial Sampling Design by SSA and Evaluations

A 100 m × 100 m grid size overlaid on the study greens spaces areas, and an initial (before optimized) kriging soil Pb predictions and MKV were produced. Then, 50 to 200 random existing sample points were perturbed using 10,000 times iterations by the SSA algorithm. A new combination is generated, and the MKV values are compared with the initial sampling layout’s value. The combination is accepted if the change has improved the MKV values. The maximum perturbed sampling points were decided based on the improved MKV values. The process continued until the prediction variance became constant or higher. The best-improved MKV combination was chosen, and a kriging prediction map and sampling distributions were generated. Finally, to evaluate prediction accuracy improvement, cross-validations were performed.

### 2.5. Statistical Analysis Software and Tools

Spatial sampling optimization and descriptive statistics were performed using the R Statistical Software (version 4.0.2) [60,61]. The spatial clusters and outliers of existing data sets were analyzed using the software GeoDa (version 1.14.0) [62]. Arc GIS (10.4 version) is also used to produce the kriging prediction maps. 

## 3. Results and Discussion

### 3.1. Mean Concentrations and Summary Statistics of Potentially Toxic Elements

The summary statistics and mean concentration of the five PTE in urban green space soils are indicated in Table 1. The highest and lowest mean concentrations were found for Zn, and Cd, respectively. The soil mean background values in Shanghai [63] and China [64] are used as reference values to compare the present study’s values. Compared to Shanghai’s background values, the mean concentrations of PTE in urban green spaces soil are much higher, except for Cd and Cr. All measured mean values exceed China’s reference values (Table 1). Pb, Cu, and Zn concentrations were 1.45, 1.20, 1.56 times the background value of Shanghai, respectively, and 1.57, 1.66, 1.91 times the mean background values in China. The higher values of PTE in the soil in comparison to background values indicate there is a soil pollution issue in the study areas.

Similarly, the coefficients of variation (CV, %) for Pb, Cu, Zn, and Cd were higher, meaning more significant variations among the urban green spaces soils (Table 1). The high CV of Pb, Cu, Zn, and Cd suggests soil pollution sources in urban green spaces are from anthropogenic sources [65]. On the contrary, the Cr CV is low, which means both natural and anthropogenic factors govern its spatial distribution. The lower CV value of Cr is consistent with many other studies [66,67,68].

The present study is consistent with the previous findings on the park and roadside green spaces in Shanghai [69], but inconsistent with results found on road-greenbelts, except for Pb [70]. The average values of Zn and Cr were significantly higher than the values reported in the western city of Urumqi in China [71]. The mean concentrations of the majority of the five pollutants considered in our study were lower than those found in studies that reported about ten years ago in roadside soil, dust, and sediment in eastern cities in China, including Shanghai [46], Guangzhou [72], and Hangzhou [73] (Table 2). 

Compared to the average concentrations in worldwide studies, Pb, Cu, and Zn values of our study were much lower than reported values from Spain, Mexico, Turkey, Sweden, and Cuba, but Cr concentration was much higher than the study from Turkey and Sweden (Table 2). In this study, the Cr concentration value was 2.7 and 5.2 times higher than the concentrations values found from Sweden, and Turkey, respectively (Table 2). For all investigated pollutants, the mean concentration values were higher than those observed in the City of Pensacola, USA (Table 2). The differences in results between this study and other studies could be due to the test method, level of urbanization in the city, the management strategies on urban green space soils [8], and the sources for variation of PTE [7], such as emissions from industry and transportation [10], smelting, manufacturing, mining, and coal-burning [11].

### 3.2. Optimization Strategy and Evaluation of Existing Monitoring Points 

The spatial interpolation in kriging is undertaken by accounting for the following assumptions [49]: (1) Data with a normal distribution, (2) data are stationary, and (3) data fit a variogram and spatial autocorrelation. Prior to carrying out the optimization strategy and the evaluation of prediction accuracy, these assumptions should be assessed and evaluated. 

#### 3.2.1. Spatial Patterns of Existing Monitoring Points 

The spatial patterns and distribution of each PTE are shown in Table 3. All variables revealed a clustered spatial distribution with a statistical significance (*p* value < 0.01) and a positive spatial autocorrelation in the existing data sets. The most clustered positive spatial autocorrelation pattern was observed for Pb and Cd (Table 3). Global Moran’s I Index values confirmed that the spatial points are clustered and non-randomness. Similarly, the kurtosis and skewness values for all pollutants, except Cr, were higher, which means the data are not normally distributed (Table 3). The higher Kurtosis values showed many outlier data sets, and the majority of them are clustered at relatively low values. However, it does not state which spatial location features are spatial clustering [80]. Spatial outliers or local outliers are neighboring values that are spatially located at a certain distance [81]. Local Indicators of Spatial Association (LISA), known as Anselin’s Local Moran’s I, were used to visualize and identify the degree of spatial instability and outliers of the given data set [55].

**Figure 2:**
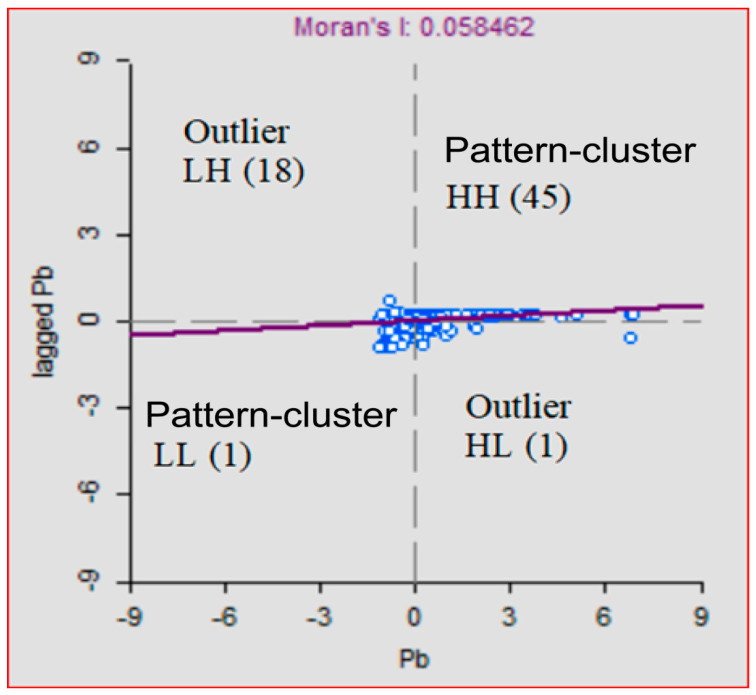
Univariate Local Moran’s I scatter plots at 12,905 m threshold distance of soil Pb.

The results of univariate Local Moran’s I scatter plots of the four PTE in the soil at 12905 m threshold distance divided into four association neighborhood layouts are indicated in the supporting data files, Figure 1. The upper right quadrant (high values above the mean surrounded by high values, HH), the lower left (low values surrounded by low values, LL); the upper left (low values surrounded by high Values, LH); and the lower right (high values surrounded by low values, HL). Spatial outlier values that include HL and LH values and spatial clusters that include HH and LL values are also indicated. For example, for soil Pb data sets, a 45 feature has neighboring features with values above the mean surrounded by HH values, and one features surrounded by LL values, which is the part of a cluster or pattern data set (Figure 2). In contrast, 19 data points have nearby features with different values (low high and high low), and this feature is a spatial outlier. Spatial outliers are the values that are different from the values recorded in their surrounding location, while spatial patterns often exhibit spatial continuity and autocorrelation with nearby samples [81]. These spatial outliers influence the spatial structure modeling and prediction of soil pollutant concentrations in urban green spaces. Therefore, the outliers were excluded, and data were transformed before the optimization strategy was undertaken. 

#### 3.2.2. Spatial Structures and Dependency 

The theoretical Semivariogram models are used to kriging interpolation and optimizing the existing points. The best-fitting Semivariogram models were selected based on root mean square error (RMSE), average standard error (ASE), and root mean square standardized error (RMSSE) values, indicated in Table 4. The best-fitted model is considered to be the one with the smallest value of RMSE, the absolute values of mean errors near to zero, the mean square error (MSE) near zero, and the RMSSE closest to 1 [82]. Based on these criteria, the fitted semivariograms models for each soil element are summarized in Table 5. The best-fit spatial model of Pb and Cr was spherical, whereas Zn and Cu were best-fitted using the Gaussian model. The Cd was fitted with the exponential model. In the semivariograms, the nugget values represent the variability of the measured variables at a certain distance. The spatial dependence and variation of soil properties can be categorized based on the Nugget/Sill ratio values. Suppose the Nugget/Sill ratio is less than 25%, between 25% and 75%, and greater than 75%, the variable has strong, moderate, and weak spatial dependence [83], respectively. All studied elements had a moderate-to-strong spatial dependency, and fit the assumptions around spatial autocorrelation (Table 5). The Nugget/Sill ratio also indicated predominant sources or soil PTE factors, either natural or anthropogenic factors. Strong spatial dependence can be attributed to intrinsic factors, and weak spatial dependence can be attributed to extrinsic factors [83].

#### 3.2.3. Prediction Accuracy Improvement by Optimization Strategy 

**Figure 3:**
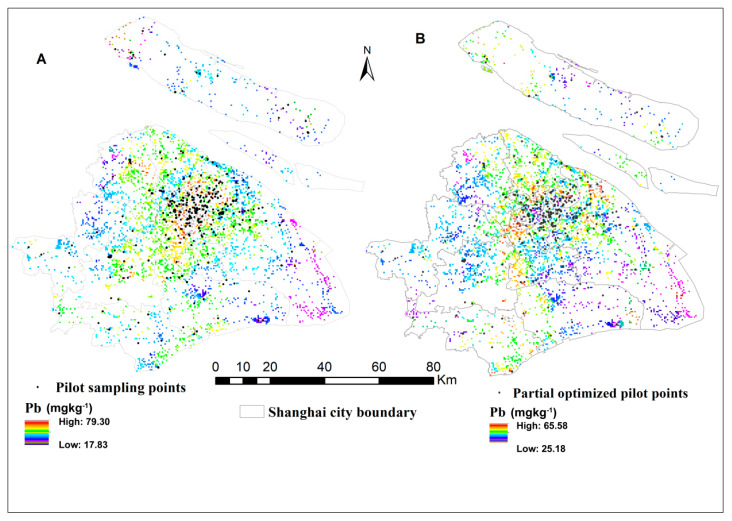
The initial sampling (**A**) and optimized (**B**) design Soil Pb concentration mapping on urban green spaces.

A kriging interpolation surface of the study green spaces soil before optimized hereafter refers to the initial sampling design shows a predicted Pb MKV of 131.74 mg kg^−1^. The kriging concentration of Pb in the initial sampling design displays spatial heterogeneity with a high prediction hotspot, which is located in the high clustered sampling points and low concentration at the edge segment, since these are the sparse and lacking in sampled areas (Figure 3a). It is also clearly noted that there are many non-sampled green spaces areas at the initial sampling design, which leads to high spatial prediction variance (131.74 mg kg^−1^). In this study, the MKV as the objective function was used in the SSA algorithm to optimize the initial sampling design [84,85]. Each SSA iteration step only involves moving one random sampling point, and the row and column of the covariance matrix are changed. As Figure 3b shows, after optimization, soil Pb sampling points were placed with a better uniformity over the study area than the initial sampling design. 

The MKV values also were calculated after the initial sampling design is perturbed by SSA (10,000 iterations). The initial soil Pb MKV (131.7 mg kg^−1^) decreased to 128.9 mg kg^−1^ under 50 random spatial samplings perturbed and 102.3 mg kg^−1^ by 200 random spatial samples perturbed (Table 6). This means the existing soil Pb sampling points captured 22.4% of the total kriged variance improvement and increased the accuracy of un-sampled green spaces without extra sampling points.

**Figure 4:**
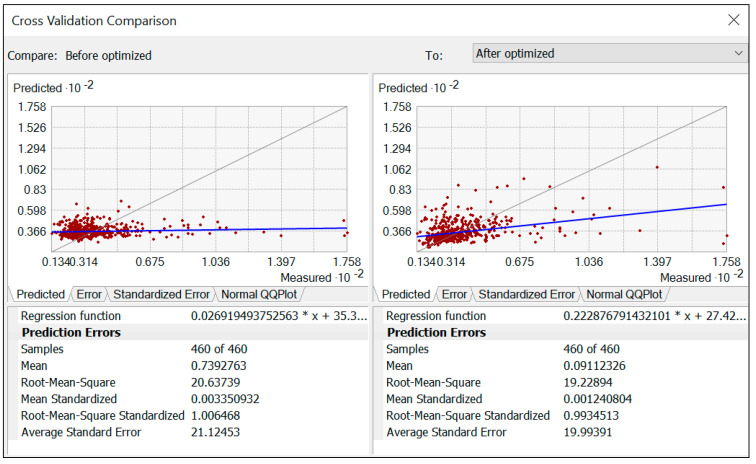
Prediction error cross-validation comparison before and after the original sampling configuration optimized by using SSA.

To evaluate the prediction accuracy and improvements in the initial sampling design, we performed a cross-validation comparison based on prediction RMSE, RMSSE, and ASE (Figure 4). The values identified for RMSE, RMSSE, and ASE of 20.63, 1.006, and 21.12, respectively, before the initial sampling design was optimized; the values were 19.22, 0.99, 19.99, respectively, after the initial sampling design optimized by SSA. The better prediction accuracy could be found in the smaller values of RMSE, the closer values of RMSE with ASE, and the values of RMSSE approximate to one (Figure 4). In contrast, the value of RMSSE is higher than one for the initial sampling design, which explains the underestimation of the variability of soil Pb predictions on green spaces soil. Figure 3a also shows a higher variability of soil Pb predication concentration in the study areas by comparing the optimized sampling configuration. Many studies confirmed that the initials sampling design samples, optimized by SSA methods, provided closer prediction results to the actual value and the lowest value of mean-variance of spatial prediction [84,86,87,88,89,90]. 

## 4. Conclusions

The current work has been carried out to investigate the five potentially toxic element concentrations and identify a method to improve prediction accuracy in the non-sampled locations in urban green space soils. The mean concentrations of five pollutants in urban green areas are much higher than Shanghai’s background values, except for Cd and Cr. However, all measured values exceed the mean reference values in China. The concentrations of Pb, Cu, and Zn were 1.45, 1.2, 1.56 times the background value of Shanghai, respectively, and 1.57, 1.66, 1.91 times the background values of China, respectively. The higher values, in comparison to the background values, may indicate the presence of soil pollution in the study areas. Similarly, the higher CV means more significant variation exists among urban green spaces soils. 

The second objective was to improve the prediction values of non-sampled locations by optimized limited sampling points in the SSA algorism. As a result, an improvement in prediction accuracy by 22.4% was found for spatial prediction in non-sampled locations. Similarly, the lower mean-variance values of spatial prediction were comparable to those the initial sampling design. Therefore, this optimization approach ensures good quality of soil pollution predictions without deleting or adding monitoring points.

## Figures and Tables

**Table 1:** Description statistics of PTE in urban green spaces soil (mg kg^−1^).

PTE	Mean	Median	Range Values	SD	CV (%)	Background Values of Shanghai *	Background Values of China **
Pb	36.96	31.70	13.41–175.8	20.20	54.66	25.47	23.50
Cu	34.41	30.27	10.09–225.4	19.04	55.32	28.59	20.70
Zn	130.3	113.6	49.15–1098	84.83	65.10	83.68	68.00
Cr	73.09	73.20	38.24–143.2	10.76	14.73	75.00	57.30
Cd	0.21	0.17	0.06–3.68	0.21	100.50	0.13	0.08

* [63], ** [64], CV = Coefficients of variation, SD = Standard Deviation. PTE = potentially toxic elements.

**Table 2:** PTE mean concentrations level (mg kg^−1^) of sampling of global urban green space areas.

Study Areas	Pb	Cu	Zn	Cr	Cd	Reference
Parks of Seville, Spain	161.0	72.00	210.0	75.00	-	[74]
Mexico City, Mexico	82.00	54.00	219.0	-	116.0	[75]
Konya Park, Turkey	289.4	427.4	289.8	14.0	21.0	[76]
Stockholm, Sweden	104.0	47.0	157.0	27.0	0.43	[77]
Tunas City, Cuba	42.0	94.0	199.0	97.0	-	[78]
Pensacola, USA	23.98	6.26	33.22	9.01	0.13	[79]
Urumqi, China	43.22	42.54	94.79	30.97	0.71	[71]
Guangzhou, China	240.0	176.0	586.0	78.8	2.41	[72]
Hangzhou, China	202.1	116.0	321.4	51.25	1.59	[73]
Shanghai, China	70.69	59.25	301.4	107.9	0.52	[46]
Shanghai, China	36.96	34.40	130.3	73.09	0.21	This study

Note: - = not data available. PTE = potentially toxic elements.

**Table 3:** Global Moran’s I Summary of statics for an existing data set of PTE in green space areas.

Variables	Moran’s I	Variance	Z-Score	*p*-Value	Distribution	Skewness	Kurtosis
Pb	0.159968	0.000312	9.178694	0.000000	Clustered	3.31	14.97
Cu	0.134803	0.000403	6.824677	0.000000		5.01	35.06
Zn	0.134243	0.00028	8.143643	0.000000		6.77	55.94
Cr	0.196636	0.000428	9.614280	0.000000		1.43	8.41
Cd	0.057502	0.000286	3.530263	0.000415		10.01	130.23

PTE = potentially toxic elements.

**Table 4:** Kriging prediction errors of interpolation by the ordinary kriging method.

PTE	Mean Error	RMSE	MSE	ASE	RMSSE
Pb	0.091	19.22	0.001	19.99	0.993
CU	0.295	18.52	0.009	18.93	1.120
Zn	0.422	81.35	0.002	104.43	0.828
Cr	0.004	9.89	−0.002	11.06	0.898
Cd	0.000	0.21	0.001	0.22	0.994

RMSE = root mean square error, RMSSE= root mean square standardized error, MSE = mean standardized error, ASE = average standard error, PTE = potentially toxic elements.

**Table 5:** Theoretical fitting semivariograms models and spatial dependency.

PTE	Model	Nugget (C0)	Partial Sill (C)	Sill (C0 + C)	Range (m)	Nugget Ratio % C0/(C0 + C)	Spatial Dependency
Pb	Spherical	0.047	0.133	0.18	2263.30	26.11	Moderate
Cu	Gaussian	0.053	0.128	0.181	2597.00	29.28	Moderate
Zn	Gaussian	0.000	0.141	0.141	213.98	0.00	Strong
Cr	Spherical	0.000	0.015	0.015	120.48	0.00	strong
Cd	Exponential	0.000	0.238	0.238	140.93	0.00	Strong

PTE = potentially toxic elements.

**Table 6:** The improvement MKV after the initial sampling design was perturbed by SSA.

Numbers of Points Perturbed	Soil Pb MKV (mg kg^−1^)	Improvement MKV (%)
50	128.9	2.16
100	118.2	10.25
150	109.1	17.16
200	102.3	22.36

## Data Availability

The data presented in this study are available on request from the corresponding author.
